# Shifts in floristic composition and structure in Australian rangelands

**DOI:** 10.1371/journal.pone.0278833

**Published:** 2022-12-14

**Authors:** Zdravko Baruch, Greg Guerin, Irene Martín-Forés, Samantha Munroe, Ben Sparrow, Andrew J. Lowe

**Affiliations:** 1 School of Biological Sciences, University of Adelaide, Adelaide, South Australia; 2 Terrestrial Ecosystem Research Network (TERN), Australia; Feroze Gandhi Degree College, INDIA

## Abstract

Monitoring shifts in vegetation composition over time is essential for tracking biodiversity changes and for designing ecosystem management strategies. In Australia, the Terrestrial Ecosystem Research Network (TERN) provides a continent-wide network of monitoring sites (AusPlots) that can be used to assess the shifts in vegetation composition and structure of Australian Major Vegetation Groups (MVGs). Here we use time-series site data to quantify the extent and rate of MVG shifts between repeat visits and to recommend the most appropriate sampling frequency for specific MVGs. The research area spans a ~1,500 km latitudinal gradient within south/central Australia from arid rangelands in the north to Mediterranean vegetation in the south. The standardized AusPlots protocol was employed to repeatedly survey 103 one-hectare plots, assessed between 2011 and 2019. Floristic and growth form dissimilarities between visits were calculated with distance metrics and then regressed against survey interval. Multivariate ordination was used to explore temporal floristic shifts. Rank-dominance curves were used to display variations in species’ importance. Between repeated visits, sites exhibited high variability for all vegetation parameters and trajectories. However, several trends emerged: (a) Species composition moved away from baseline linearly with intervals between surveys. (b) The rate of species turnover was approximately double in communities that are herbaceous versus woody-dominated. (c) Species abundances and growth forms shift at different speeds. All floristic and structural metrics shifted between re-visits, with varying magnitude and speed, but herbaceous-dominated plots showed higher floristic dynamism. Although the expanse, logistics, and the short time between visits constrained our analysis and interpretation, our results suggest that shorter revisit intervals may be appropriate for herbaceous compared to woody systems to track change most efficiently.

## Introduction

In Australia, rangelands are defined as land supporting low-intensity and extensive livestock grazing. They extend over 81% of the continent, from the northern monsoonal savannas to the southern temperate lands that bound the central deserts [1,2]. Rangelands contain a large portion of Australian biodiversity [1,3–5] which is exposed to increasing land conversion, invasive weeds, grazing by feral and domestic herbivores, changed fire regimes, and the cumulative interaction of these stressors, that cause biodiversity loss and ecosystem service deterioration [6,7].

Understanding the dynamics and sensitivity of rangeland ecosystems and forecasting their response to future climates is critical for biodiversity conservation, ecosystem management and for identifying the direction of these changes [8–10]. In this task, detecting and measuring shifts in vegetation cover and composition over space and time is essential. Australian rangeland vegetation has been surveyed over time mostly for pastoral purposes, and usually at a local scale [11–14]. A large inventory of plot surveys, with spatial and temporal replication, is now available through the Terrestrial Ecosystem Research Network program (TERN - https://www.tern.org.au) and it is opportune to assess the changes that this monitoring program can capture. Whilst the TERN program has continental coverage, we focus our analysis here on vegetation shifts of arid rangelands of central Australia and their gradual transition to southern Mediterranean-type woodlands. Both landscapes are characterized by old, geologically diverse substrates, with mostly infertile soils with highly variable rainfall. This combination of abiotic conditions, modulated by fire and human intervention, underpins the vastly heterogeneous and fragile vegetation types [15–17].

Our multiannual research encompasses six major vegetation types from ten Australian Bioregions [IBRA, V. 7; [18,19]] and enhances previous studies in that our surveys are standardized, and vegetation structure is incorporated into the analysis. Currently, 103 one-hectare plots are established to study temporal changes in vegetation floristics and structure. For six of the Australian Major Vegetation Groups [MVGs; National Vegetation Information System [20]], we aimed here to: (a) Identify the MVGs with the highest and lowest compositional and structural variability; (b) Identify the species and growth forms responsible for the most pronounced vegetation changes and (c) Ascertain if MVGs change at similar or different rates. Based only on our surveys, we expected that as the time between visits increased, so would the differences in floristic and structural traits across all MVGs. Likewise, and due to the shorter life span of grasses and forbs, we expected shifts in herbaceous-dominated MVGs to be detectable faster than those in wooded-dominated MVGs. By quantifying differences in vegetation rates of change, appropriate intervals for monitoring can be established for each MVG [21–25]. Currently, our study is limited by the unequal number of sites within each MVG and uneven survey frequencies. Therefore, the results presented and discussed here are subjected to continuous enlargement and revision as the TERN Surveillance Network (https://www.tern.org.au/tern-observatory/tern-ecosystem-surveillance/) is expanding with new and more re-visited plots [25] and our ability to analyse change will increase significantly in the coming years.

## Methods

**Fig 1:**
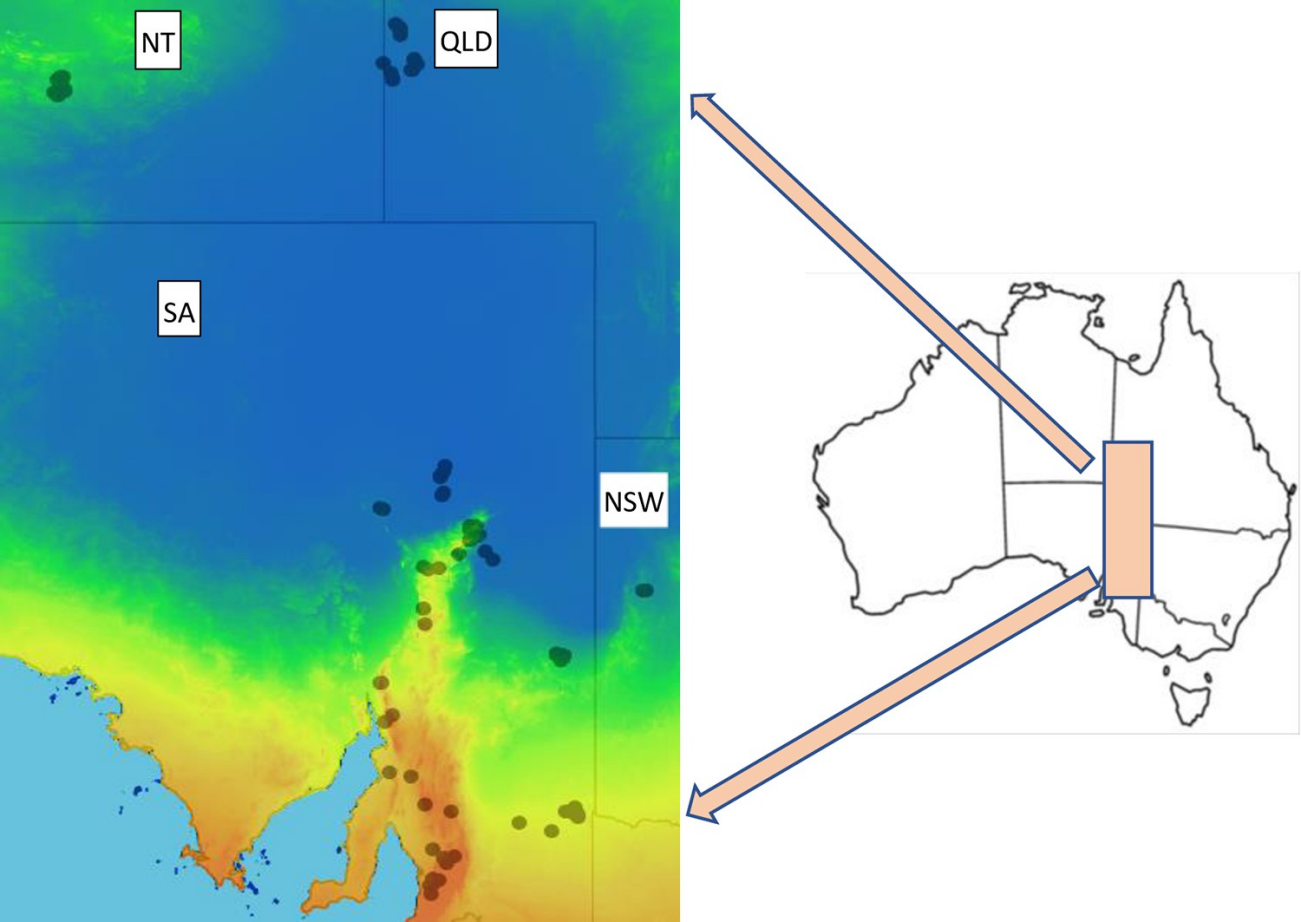
Location of the repeatedly surveyed plots. Location of the repeatedly surveyed plots within the Northern Territory (NT), Queensland (QLD), South Australia (SA), and New South Wales (NSW). Three plots from Western Australia are not shown. The background layer represents the annual mean aridity index (precipitation/evaporation) from the more arid areas in blue to the more mesic areas in orange. The Australian outline map indicates the location within the continent. Maps downloaded from the Atlas of Living Australia. https://spatial.ala.org.au. Accessed 1 November 2022. Creative Commons license CC BY 3.0 AU.

The study area was established by the plots censused two or three times in south-central Australia. It spans approximately 1,500 km from mid-continental to coastal latitudes intersecting the states of New South Wales, Northern Territory, Queensland, and South Australia (Fig 1). The northern portion of our study is arid or semi-arid and is committed to extensive livestock grazing [1]. Hot days, very low and erratic rainfall, and a large and diverse group of herbaceous and shrubby communities with generally low cover characterize this landscape. Grazing and browsing by domestic and feral herbivores and the prominent invasion of buffel grass (*Cenchrus ciliaris*) in some areas have diminished vegetation diversity by outcompeting native species and by increased frequency and intensity of fire [6].

Within the study area, we used AusPlots vegetation surveys conducted nationwide by TERN [24,26] and selected according to prerequisites and strategies detailed in [20,27]. Floristic and structural vegetation data are from 216 repeated (two or three visits) surveys between 2011 and 2019 with intervals between visits ranging from one to eight years. The plots encompass ten IBRAs and represent 14 MVGs [20] (Figs 1 and S2).

Southwards from approximately 32°S, the arid rangelands gradually change to a more humid and wooded Mediterranean-type terrain [28]. Here, Eucalypt and Mallee Woodlands mark the landscape with the characteristic signature provided by the grass-tree genus *Xanthorrhoea*. This area contains high phylogenetic diversity and endemism embracing one floristic refugium [3]. The climate is temperate and less arid with winter rains and summer droughts (S1 Table). Soil fertility, pH and carbon content are relatively high as is the soil water holding capacity [29]. This region has a far higher human population density and key threats to local vegetation are land clearing and fragmentation, fire and urban encroachment [7,30]. Throughout the study area, cycles of dry and wet years are the norm (S1 Fig).

Within the study area, we used vegetation data from AusPlots vegetation surveys conducted nationwide by TERN [24] and selected according to prerequisites and strategies detailed in [20,31]. In brief, site and plot selection was supported by ecological (e.g. stratification across representative bioregions and sampling `best on offer’ habitats), administrative, and logistic considerations (e.g. feasibility of access). Floristic and structural vegetation data are from 216 repeated (two or three visits) surveys between 2011 and 2019 with intervals between visits ranging from one to eight years. The plots encompass ten IBRAs and represent 14 MVGs [20] (Figs 1 and S2).

### Data collection

Throughout visits, surveyors matched each plot to one MVG and assessed their condition by recording disturbances such as recent grazing, flooding and fire following the AusPlots protocol [25,32]. Briefly, within one-hectare plots, species are recorded at each meter along 10 x 100m transects (1010-point intercepts, PIs) which are converted to percentage cover for each species (number of PIs/1010). Species frequency is the number of transects where the species is recorded (ranges from 1 to 10). In addition, all vascular plants in the plot were recorded and vouchered for herbarium identification.

The plot’s vegetation structure was established by the proportion of species sorted within the growth form classification defined in the Australian Soil and Land Survey Field Handbook [33]. We employed the percentage of each growth form to generate profiles (or spectra) for the structural description of plots and comparisons across visits. The percentage of plot area covered by either vegetated ground cover or bare soil was calculated as a proxy of habitat aptness for plant growth. The identity of non-native and potentially invader species in Australian rangelands was established after [34,35].

### Data management and analysis

We extracted data from re-visited plot surveys using the ‘ausplotsR’ v1.2 package (CRAN: https://CRAN.R-project.org/package=ausplotsR; latest development version and patches: https://github.com/ternaustralia/ausplotsR [31,36]. For each species and growth form, Importance Value Index (IVI) [37] (S1 Text). Unless stated otherwise, all floristic and structural analyses were based on IVI, as it provides a more balanced representation of each species and growth form standing in the plot. The variability of floristic and structural traits of plots and MVGs calls for grouping those with shared physiognomic similarities. Consequently, we characterised and grouped three major streamlined vegetation types: Grasslands, represented by the Hummock and Tussock MVGs; Shrublands, represented by the Acacia and Chenopod MVGs; and Woodlands characterized by Eucalypt and Mallee MVGs. We also merged plots as either being wooded or herbaceous.

Species diversity was assessed with standard procedures whereas the floristic difference across visits was assessed with the Sörensen (Bray-Curtis) distance and the Simpson Beta metrics [38] (S1 Text). The dissimilarity of each revisit to its baseline survey was regressed against survey interval in years using linear models. The slope and fit of the models, related to the rate of compositional change over time, were compared between MVGs grouped within grasslands, shrublands and woodlands. Annualized shifts (S1 Text) of compositional distances and ANOVAs tested for differences among MVGs. It has been We considered that more than four years between visits was a reasonable lapse for shifts to become evident [26]. Non-metric multidimensional scaling (NMDS) illustrated the extent of temporal floristic changes (S1 Text). To contrast shifts in species dominance, we fitted empirical lognormal models [39] and tested the sigma (σ) shape parameter. Changes in species dominance across visits were represented by Whittaker plots [40].

**Fig 2:**
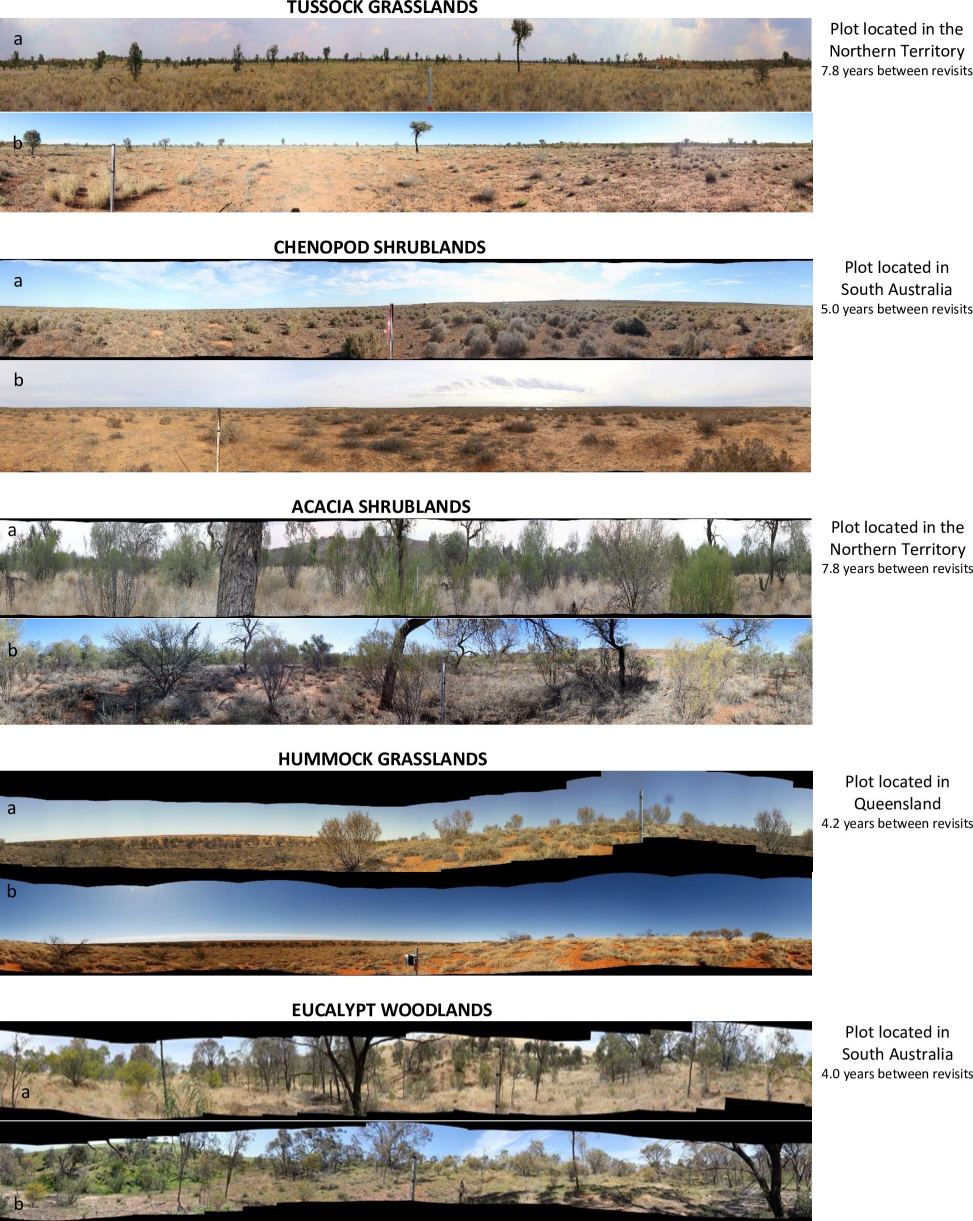
Panoramic view of plots. Panoramic view of plots. with the largest shifts in vegetation between the first (a) and last (b) visits and the interval between surveys. The panoramic photos could not be lined up precisely, but angle and depth of field were as similar as possible. Photo Credit: TERN Ecosystem Surveillance Program.

For the structural analysis, we merged the classification of growth forms [31] into eight groups (S1 Text) and we computed their respective IVIs and determined their percentage within each MVG. The percentage of total area with vegetation was also calculated. Shifts in MVGs’ structure (growth form allocation and vegetated area) over time were assessed as that for species cover described previously. In addition, a simplified scheme of vegetation structure shifts tallied the number of plots in which the proportion of wooded and herbaceous vegetation changed after more than four years from the initial visit. To illustrate structural shifts of selected MVGs, we drew growth form allocation shifts across time (S5 Fig) supported by panoramic photographs (Fig 2).

## Results

Our dataset tallied 992 species (including 40 that are non-native) from 97 families and 16 growth forms (site and species data are accessible online: University of Adelaide figshare DOI 10.25909/19252049 and DOI 10.25909/19210167). Fourteen MVGs were categorised by the surveying team. Plots from Acacia Shrublands, and Eucalypt and Mallee Woodlands were the most numerous (S2 Fig).

### Species composition

**Fig 3:**
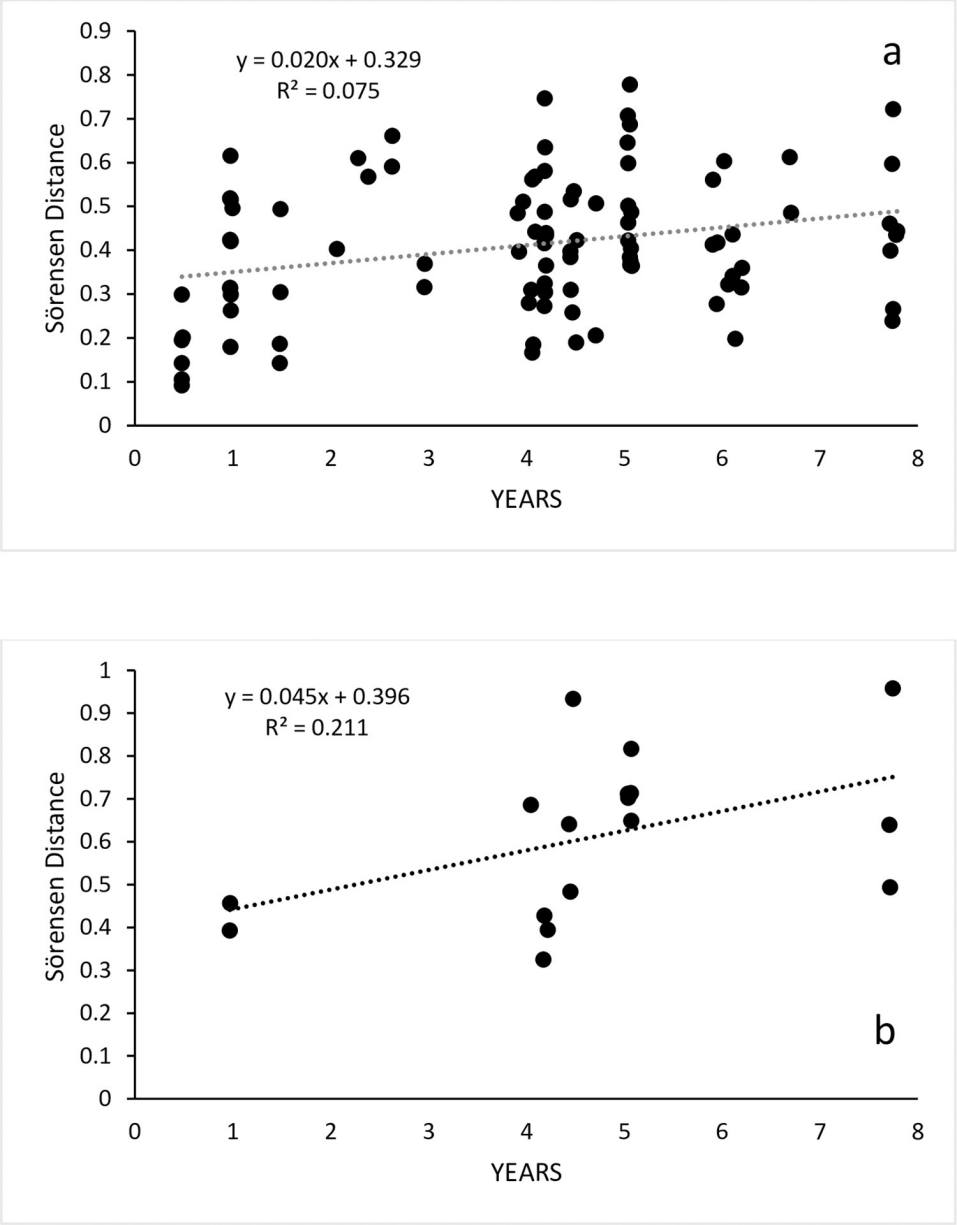
Sörensen (Bray-Curtis) distances. Sörensen (Bray-Curtis) distances. from first (year 0) to last survey, with regression line and statistics. (a) plots dominated by woody species (b) plots dominated by herbaceous species.

Species richness and diversity were highly variable at the plot scale, and this translated to large variances even within MVGs (S2 Table). Hummock Grasslands were the most divergent due to their relatively low species richness and diversity as well as their higher dominance by few species and with relatively low β-diversity (S2 Table). Across all MVGs, neither species richness nor diversity varied according to time between visits. Sörensen dissimilarity increased with time between visits and this trend was stronger for the herbaceous than for the wooded MVGs, with a two-fold difference in linear slope (Fig 3) In contrast, the Simpson Beta metric (which calculates plot differences excluding species richness) was unrelated to time between visits (all MVG slopes below 0.01). Although our survey period included dry and wet years, we were unable to detect consistently associated floristic responses.

**Fig 4:**
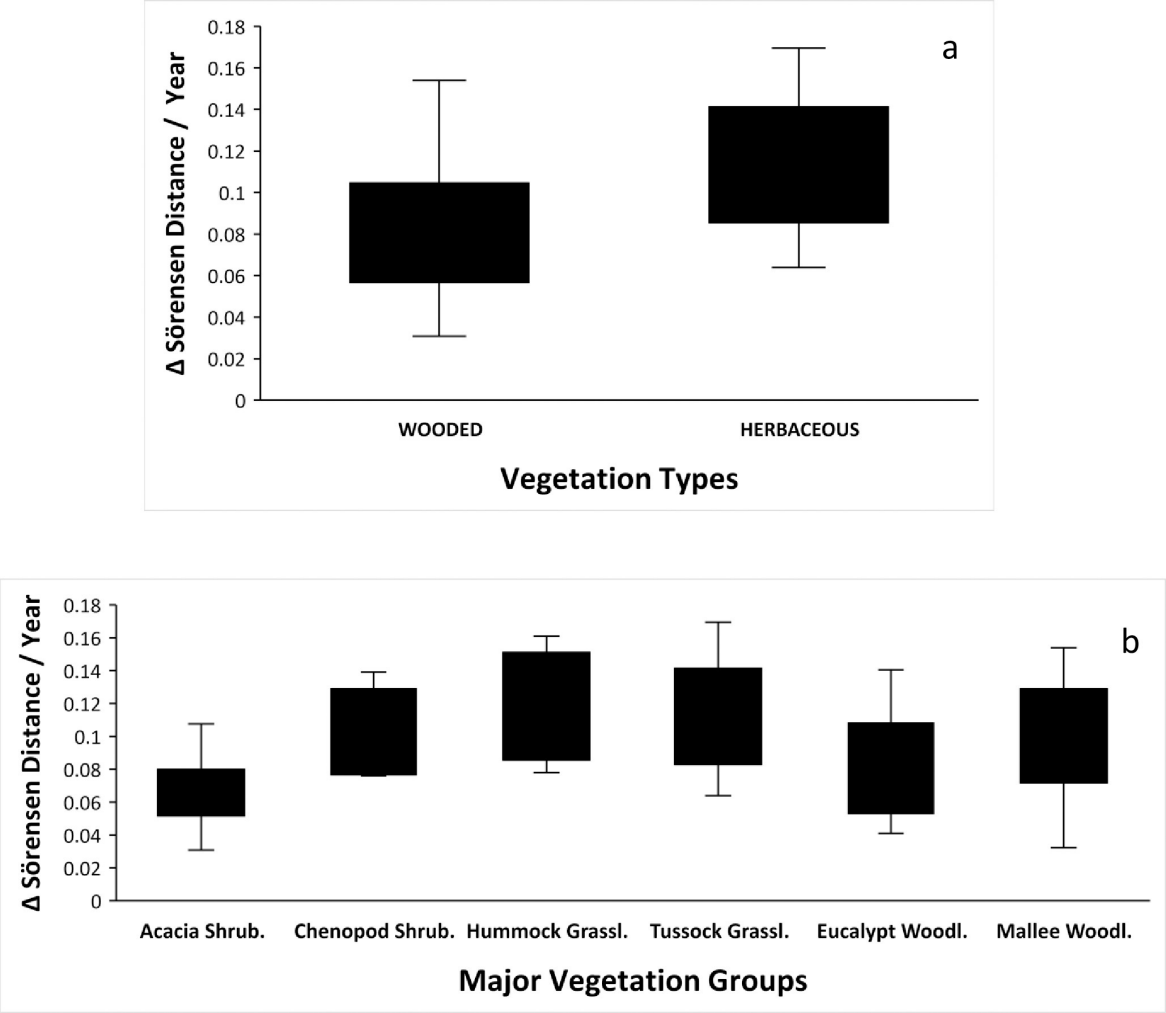
Annualized floristic Sörensen distances. Annualized floristic Sörensen distances for revisits after more than 4 years. (a) Contrast between Wooded and Herbaceous vegetation types. ANOVA F_(1,56)_ = 11.27; P = 0.001. (b) Contrast between six Major Vegetation Groups. Global ANOVA F_(5,53)_ = 2.90; P = 0.022. Only Acacia Shrublands and Tussock Grasslands are statistically different at P = 0.025.

Annualised compositional changes for grouped MVGs were faster in the herbaceous ones (P = 0.001) (Fig 4A). Although the global ANOVA showed significant differences among individual MVGs (P = 0.022) and fastest changes were for Tussock and Hummock Grasslands, only Acacia Shrublands and Tussock grasslands were statistically different (P = 0.025) (Fig 4B). Visual appraisal of non-metric multidimensional scaling ordination confirmed that Tussock and Hummock Grassland plots displayed some of the largest floristic shifts whereas most of the Eucalypt Woodland plots displayed the smallest shifts relative to other plots (S3 Fig).

Plots from all MVGs showed a mix of increased and decreased species dominance responses across visits (Table 1). Increased dominance, evidenced by lower σ values on the last visit, was more accentuated in Acacia Shrubland and Eucalypt Woodland plots, and less in Chenopod Shrublands and Hummock Grasslands (Table 1). For illustration, contrasting arrays of rank-dominance curves across visits are shown in S4 Fig.

**Table 1:** Log-normal sigma for rank-dominance curves.

MVG	Increased σ	Decreased σ	Increased σ	Decreased σ	ANOVA	
	n	n	Mean ± SE	Mean ± SE	F	P
Acacia Shrublands	4	8	0.085 ± 0.037	0.108 ± 0.026	18.59	0.002
Chenopod Shrublands	3	5	0.170 ± 0.120	0.168 ± 0.093	4.95	0.068
Eucalypt Woodlands	11	17	0.110 ± 0.036	0.177 ± 0.029	39.89	<0.001
Mallee Woodlands	6	7	0.128 ± 0.027	0.089 ± 0.025	34.65	<0.001
Hummock Grasslands	2	3	0.080 ± 0.046	0.091 ± 0.048	8.10	0.065
Tussock Grasslands	3	4	0.195 ± 0.089	0.160 ± 0.077	9.15	0.029

Number of plots, means and ANOVA of sigma (σ) values (as modelled by log-normal curves) that shifted from the first to the last visit. Higher sigma values indicate decreased dominance; lower values indicate increased dominance. ANOVA tested for differences between increased and decreased σ.

### Vegetation structure

The Sörensen distance metric distinguished growth form allocation shifts across time elapsed from baseline visit. However, only Shrubland plots displayed a moderate increase in growth form shifts across time (Regression equations and coefficients: Grasslands: y = 0.0022x + 0.185; n = 18; R^2^ = 0.0026. Shrublands: y = 0.0308x + 0.0697; n = 32; R^2^ = 0.159. Woodlands: 0.0065x + 0.1389; n = 53; R^2^ = 0.0148). The contrast in growth form allocation dynamics is illustrated by one Hummock Grassland plot with only relatively minor changes across time against one Acacia Shrubland plot that was more dynamic regarding changes in vegetated area and cover of tussock (S5 Fig).

**Fig 5:**
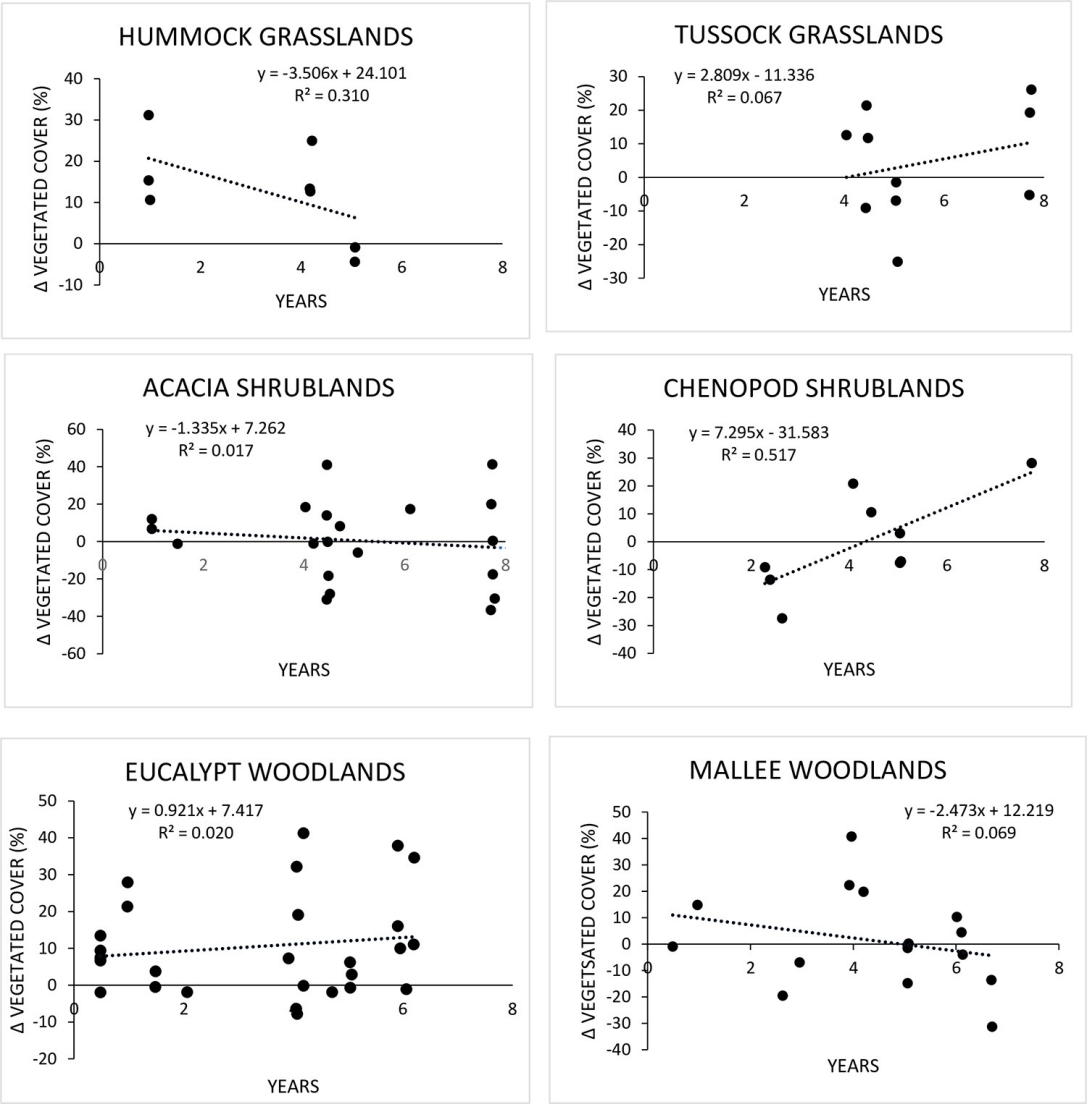
Shifts in vegetated cover. Shifts in vegetated cover (%) after the first (baseline, 0 years) visit.

Wooded, herbaceous and soil cover shifted the least in Hummock and Tussock grasslands after more than four years from baseline visit (Table 2). Notably, Shrublands had the highest number of plots with a decrease in wooded cover (88.2%; Table 2) supporting the result obtained by the Sörensen distance metric approach. Among Woodland plots, wooded vegetation cover varied little, but understory herbaceous vegetation and bare soil did change appreciably and appeared to be comparable in extent (Table 2). The proportion of vegetated area that shifted after the baseline visit offers additional information on plot structure changes. Only Hummock Grassland and Chenopod Shrubland plots markedly shifted vegetated area across visits as gauged by the slope of the linear regression. The former decreased and the later increased in vegetated area (Fig 5).

**Table 2:** Number of plots with shifting wooded, herbaceous, or bare soil cover (%).

Major Vegetation Groups		NUMBER OF PLOTS			
	SHIFTS	Wooded Vegetation	Herbaceous Vegetation	Bare soil	Total Plots
Hummock & Tussock Grasslands	Increased	5 (41.7)	4 (33.3)	6 (50.0)	
	Decreased	5 (41.7)	8 (66.6)	6 (50.0)	12
	Unchanged	2 (16.6)	0 (0)	0 (0)	
Acacia & Other Shrublands	Increased	2 (11.7)	6 (35.3)	10(58.8)	
	Decreased	15 (88.3)	11 (64.7)	7 (41.2)	17
	Unchanged	0 (0)	0 (0)	0 (0)	
Eucalypt & Mallee Woodlands	Increased	19 (54.3)	22 (62.8)	11(31.4)	
	Decreased	15 (42.9)	10 (28.6)	20(57.1)	35
	Unchanged	1 (2.8)	3 (8.6)	4 (11.5)	

Plots grouped by MVGs. Wooded vegetation includes shrubs and trees. Herbaceous vegetation includes hummock and tussock grasses. Results presented are only for plots visited after more than four years from baseline. Percentages of shifting plots shown in parentheses.

## Discussion

The 1,500 km long latitudinal research area encompasses large physical gradients and varied external stressors (fire, grazing and invasive species) that shape the composition and structure of local vegetation and influence their temporal shifts. Our objective to understand the complex rangeland vegetation dynamics is challenging due to the current limited and uneven number of surveyed sites and the non-uniform survey frequencies unavoidable in this type of continental study [22]. Nevertheless, at this stage of research, we found that: (a) All re-visited plots were distinct from their baseline counterparts, with floristic and structural traits shifting substantially over time. (b) MVGs displayed distinct responses across time that are contingent on the analytical tools employed. (c) MVGs shifted at different rates with higher dynamism in the herbaceous-dominated ones and (d) Wooded and herbaceous-dominated MVGs diverge the most in floristic and structural shifts.

The results presented and discussed here are preliminary. Ongoing research by the Ausplots Surveillance Team on more plots and longer time spans between visits will reveal differences in MVGs shifts with more confidence. Nevertheless, since we surveyed across diverse time spans between visits, our present results and, in the absence of stochastic events, imply that herbaceous dominated plots would require more frequent surveys than wooded dominated plots for detecting community shifts. Also, our analytical approach asserts the value of the MVG classification scheme as a useful analytical tool.

### Species composition

The Sörensen distance between initial and final vegetation composition detected a positive association with time between surveys which was stronger for the herbaceous Hummock and Tussock Grasslands than for Shrublands and Woodlands. Tussock Grasslands displayed the fastest shifts. This response was possibly related to the mostly shorter life spans and shallower roots of grasses and forbs that are more vulnerable to years of drought that occurred throughout the study (discussed below). The ordination of plots visited three times shows multi-directional floristic changes. Additional surveys on the same plots for longer periods will improve prediction of trajectories.

Our chronological sequence of rank curves, comparable to the so-called rank clock [39], captures and summarizes floristic dynamics. The durability of shifts in species rank are early signs of vegetation stability and trajectory [39]. Reordering of species rank across time from lax to accentuated dominance and vice versa occurs in plots of all MVGs and are a typical attribute of community assembly caused by the interaction of biophysical and demographic factors with plant functional traits. Plots from all MVGs displayed increased dominance across time and this response was more accentuated in Acacia Shrublands and Eucalypt Woodland plots and less in Chenopod Shrublands and Hummock Grasslands. In non-experimental settings, this type of result is difficult to explain although ours matches one reported from a desert in New Mexico (USA) [39] in that rank shifts are more noticeable in grasslands than in shrubland vegetation. These rank-dominance descriptions and conclusions should be appraised with caution, as it might be possible that the timing of the survey interacted with the phenology of short-lived species and opportunistic invader plants which could be tested through an independent analysis.

As rainfall totals and multiannual rainfall sequences are the main environmental filter and ecosystem driver in Australian rangelands [12,21,23,29], and because our surveys spanned two clearly discernible episodes of dry years (rainfall was 64% and 21% lower than average in the arid areas), we anticipated certain vegetation responses. However, our prediction that herbaceous plots would be more responsive to dry spells, due to their mostly shallower roots and shorter life spans, was not confirmed. This could be caused by several factors. First, the comparison between dry and wet years for individual MVGs is inappropriate as this was done on sets of plots that differ in species richness and cover. Second, the seasonal timing of survey field trips was not uniform. For example, a survey after a rainy sequence of days might display more annual plants than a later survey. Third, the climatic annual variability across MVG’s varies greatly across our study area the northern section is aseasonal and the southern section is highly seasonal. Finally, the lag effect of water stored in the soil [12] as well as the water retention capacities associated with soil texture and the water use efficiency of the vegetation were unexplored. Again, more surveys across longer time spans would clarify the impact of the role of dry-year spells on vegetation dynamics.

Invasive species are one of the main drivers of vegetation changes [16] and the opportunistic buffel grass (*Cenchrus ciliaris*) is the most prominent in our arid sites [40]. Further studies derived from subsequent visits would allow studying the impact of invasive species as well as the effect of predicted increase in aridity and temperature on the responses of buffel and other C_4_ grasses.

### Vegetation structure

The fraction of vegetated area and the allocation of growth forms define and characterize the physiognomy of MVGs. Both attributes bound the resources for consumers and influence microclimate by shading and regional climate by albedo and evapotranspiration [41]. Vegetated area, or its reciprocal bare soil area, was the major and most dynamic molder of structure in our plots. Consequently, the smaller shifts in the fraction of bare soil in Woodlands sign for higher steadiness as compared to other MVGs. This could be a consequence of less aridity in the Woodland southern plots or due to tree longevity. Shifts in growth form allocation across time are also less noticeable in Grassland plots. This apparent contradiction with floristic shifts suggests that floristic shifts are likely to be faster than shifts in the growth form assemblage. However, it is also possible that species may flux in and out of the community while the structure remains unchanged. The increase in vegetated cover in chenopod shrublands during the relatively dry period across revisits is counter-intuitive but could have been driven by de-stocking. This possibility is supported by results from a fenced plot experiment in the South Australian shrublands [13]. Another facet of landscape dynamics emerges by recording the number of plots that shift the proportion of wooded and herbaceous elements across time. In this instance, Shrublands are the most dynamic MVG as its wooded component decreased in more than three-quarters of surveyed plots.

## Conclusions

All plots displayed compositional shifts that were not clearly directional nor occurring at the same rate. The degree of relative shifts of MVG plots is contingent on the traits used for assessment. Thus, floristic shifts were more pronounced in herbaceous plots, but structural shifts were more pronounced in shrubland plots.

Our analysis and interpretations were subjected to limitations of this type of field research such as expanse and travel time-cost logistic trade-offs. A related aspect is to consider if the relatively short time encompassed by this study was enough to detect permanent changes. Only future re-visits of more plots and lengthier time spans between visits will enable us to assure that the observed shifts are lasting and to determine the directionality of their trajectories. However, this task remains challenging as the increased temperature and altered rainfall patterns of future climatic changes alter the phenology of vegetation (annual cycles of greening and senescence, for example) [42] which in turn influence field cover measurements. Furthermore, the phenology shifts are expected to be more pronounced in herbaceous than in wooded vegetation.

A practical application of this study is to establish the most appropriate frequency of surveys. Although climate and disturbance contexts are critical for setting monitoring frequencies for arid vegetation, our results showed that herbaceous MVGs require more frequent re-visits than wooded ones. Although it is never possible to survey all plots and MVGs in the same year, our results could also address future surveys of the most promising plots to detect vegetation shifts.

## Supporting information

S1 Fig Rainfall from selected climatic stations. Rainfall from selected climatic stations. closest to surveyed plots. (a) Representative rainfall pattern for arid rangeland plots. (b) Typical rainfall pattern for southern Mediterranean plots. Also shown are mean rainfall, Bureau of Meteorology (www.bom.gov.au) designation ID and coordinates. Rainfall data for Alice Springs in years 2010 and 2020 are incomplete.
(TIF)

S2 Fig Number of plots of each MVG sampled. Number of plots of each MVG sampled as plot/visits across all Major Vegetation Groups (MVG). Forests were excluded from our analysis. Herbaceous-dominated MVGs are Hummock and Tussock Grasslands.
(TIF)

S3 Fig Non-Metric Scaling Ordination of plot/visits from MVGs. Colours characterize plot location: South Australia = red; Northern Territory = blue; Queensland = green; New South Wales and Western Australia = black. Arrows indicate direction of change and their length (two surveys) and area of polygons (three surveys) represent magnitude of changes.
(TIF)

S4 Fig Contrasting rank-dominance curves. For selected plots across two surveys visits. Inserted is the shape parameter sigma (σ) from the modelled lognormal curve. Species and rank are also shown.
(TIF)

S5 Fig Illustration of growth form allocation shifts. Two selected plots throughout a three-visit sequence are presented. Dates, lapses between visits and the proportion of ground covered by vegetation (VA) are also shown.
(TIF)

S1 Table Climatic parameters of Major Vegetation Groups. Mean and standard deviation of main annual climatic parameters for Major Vegetation Groups. The Aridity Index is the ratio of precipitation to potential evaporation (higher values indicate less aridity). n = number sites. Data from Harwood et al. (2016).
(DOCX)

S2 Table Floristic metrics and statistics of Major Vegetation Groups. Indicated are the number of plots (n) and species. S, E, H’ and D designate plots species richness, equity, Shannon’s and Simpson diversity indices, respectively. Vouchered species refers to their total number within plots. Maximum Importance Value Index (IVI) denotes the highest value for species in the plots. Maximum IVI denotes increased dominance. β-Diversity indices by Whittaker’s (W) and as half-changes.
(DOCX)

S1 Text Expanded information on data management and analysis. The IVI was calculated using the relative cover [(cover/summed cover for all species/growth forms) *100] and relative frequency [(frequency/summed frequency for all species/growth forms) *100] were calculated. Adding relative species cover and frequency generates the species IVI. Similarly, the IVI of each growth form was assessed by adding their relative number of species and its relative number of PIs. Species richness and Shannon and Simpson indices assess species diversity and relative dominance. The Sörensen (Bray-Curtis) distance and the Simpson Beta metrics [36], represent compositional change over time, including and excluding, respectively, differences in species richness. To contrast the rate of floristic shifts between MVGs on a common foundation, we annualised the shift of Sörensen distances by dividing them by the duration of the interval between visits and ANOVA tested for global differences among MVGs. In addition, to illustrate the extent of temporal floristic changes, plots from each MVG were ordered with the non-metric multidimensional scaling method (NMDS) representing Sörensen distances between visits as vectors [PC-Ord v7 [38]] To numerically contrast shifts in species dominance, we fitted lognormal models to the empirical species abundance distributions [39] then computed and tested the differences in the sigma (σ) shape parameter. The eight groups [31] for structural analysis are: (a) Trees (comprising Tree Mallee, Tree-Palm and similar); (b) Shrubs (including Heath Shrub, Shrub Mallee, and Acacia Shrub); (c) Sedges; (d) Hummock Grass; (e) Tussock Grass; (f) Chenopods; (g) Forbs and Herbs and (h) Others with minute abundance (Epiphyte, Fern, Rush, and Vines). Then, we computed their respective IVIs and determined their percentage within each MVG.
(DOCX)
