# Supplementary figures and images for: Shifts in floristic composition and structure in Australian rangelands
Source: PLoS One. 2022 Dec 14;17(12):e0278833. doi: 10.1371/journal.pone.0278833 (PMC9750033; doi:10.1371/journal.pone.0278833)

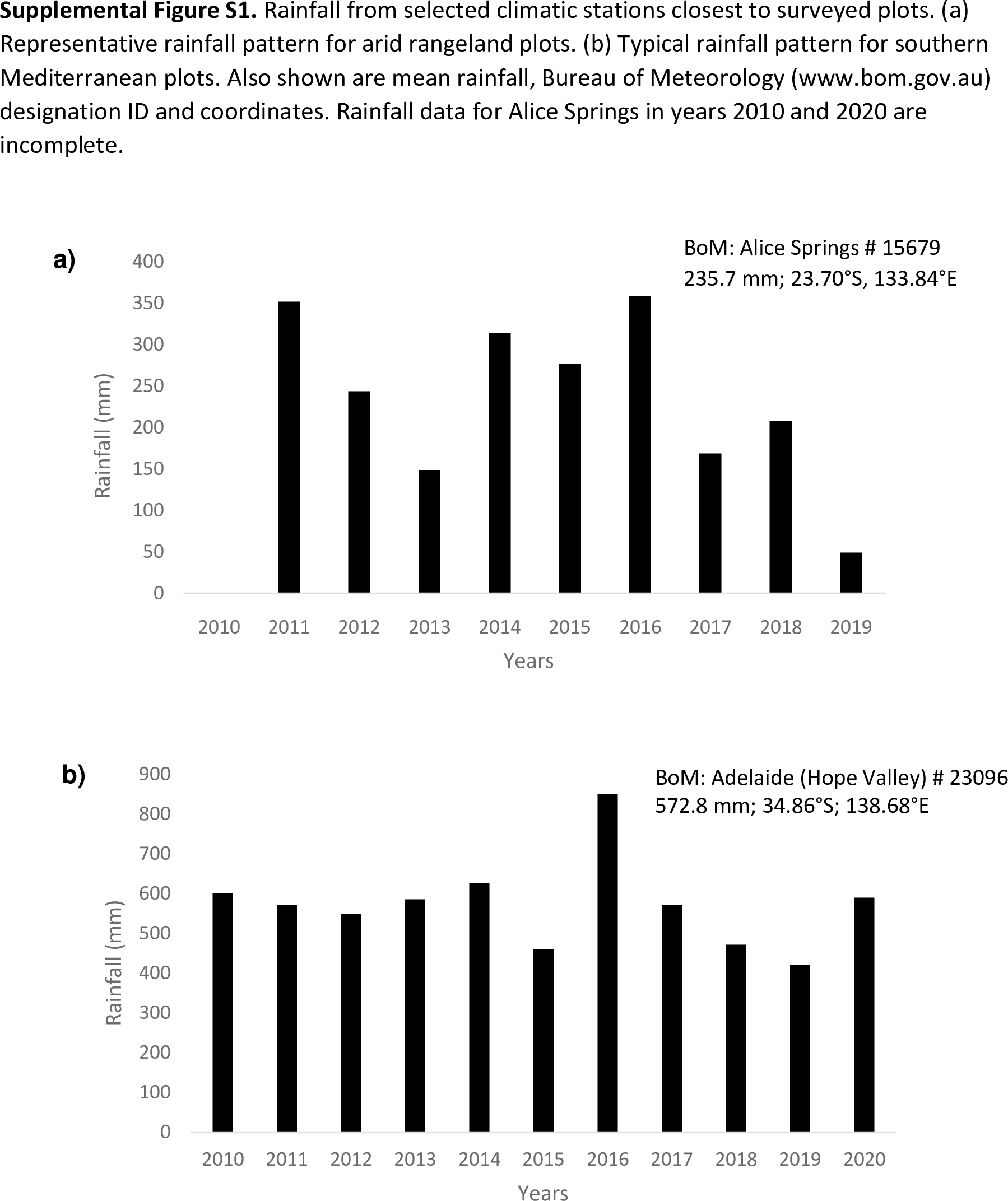

Supplement: S1 Fig — Rainfall from selected climatic stations. closest to surveyed plots. (a) Representative rainfall pattern for arid rangeland plots. (b) Typical rainfall pattern for southern Mediterranean plots. Also shown are mean rainfall, Bureau of Meteorology (www.bom.gov.au) designation ID and coordinates. Rainfall data for Alice Springs in years 2010 and 2020 are incomplete. (TIF) [file pone.0278833.s001.tif]

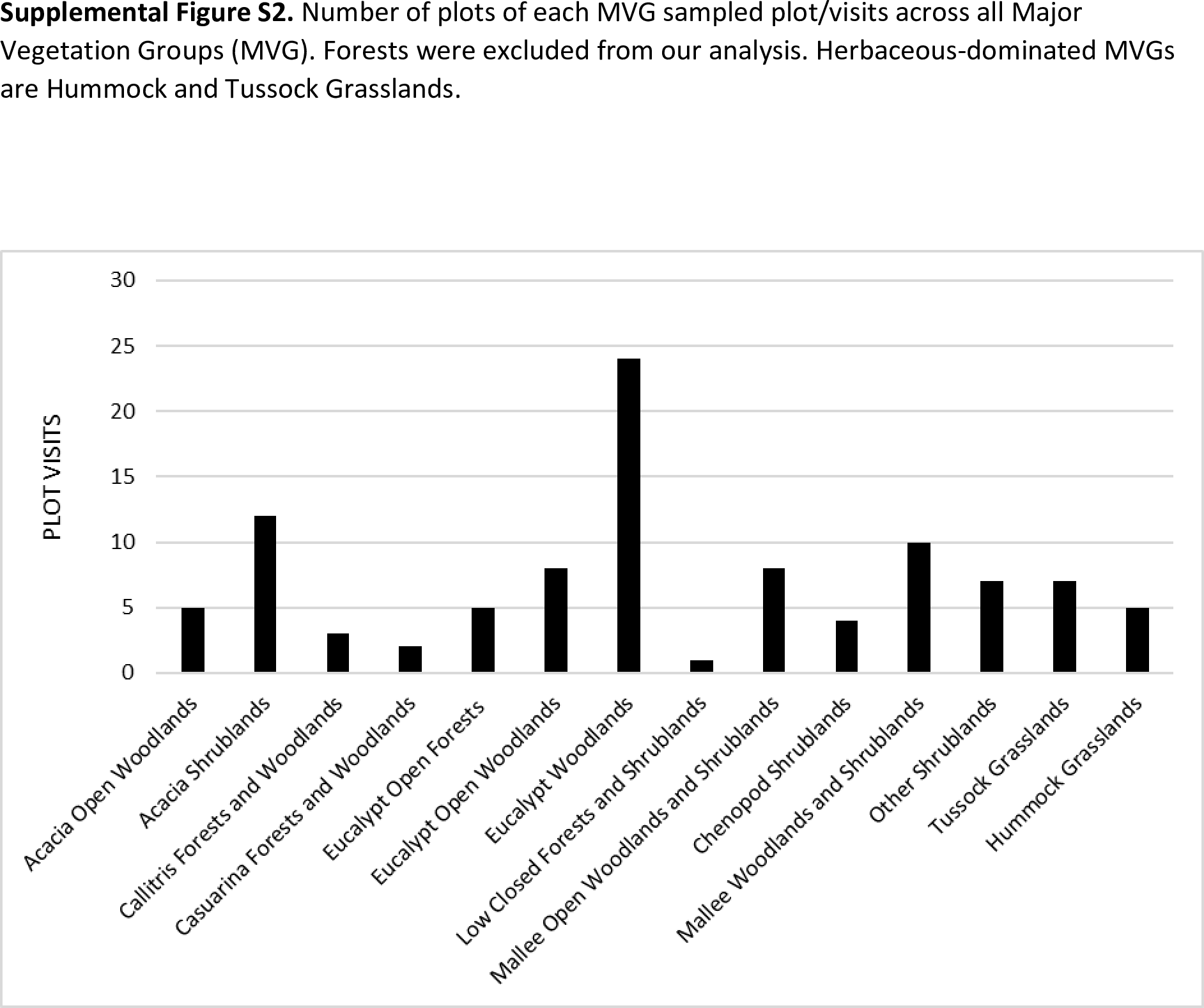

Supplement: S2 Fig — Number of plots of each MVG sampled as plot/visits across all Major Vegetation Groups (MVG). Forests were excluded from our analysis. Herbaceous-dominated MVGs are Hummock and Tussock Grasslands. (TIF) [file pone.0278833.s002.tif]

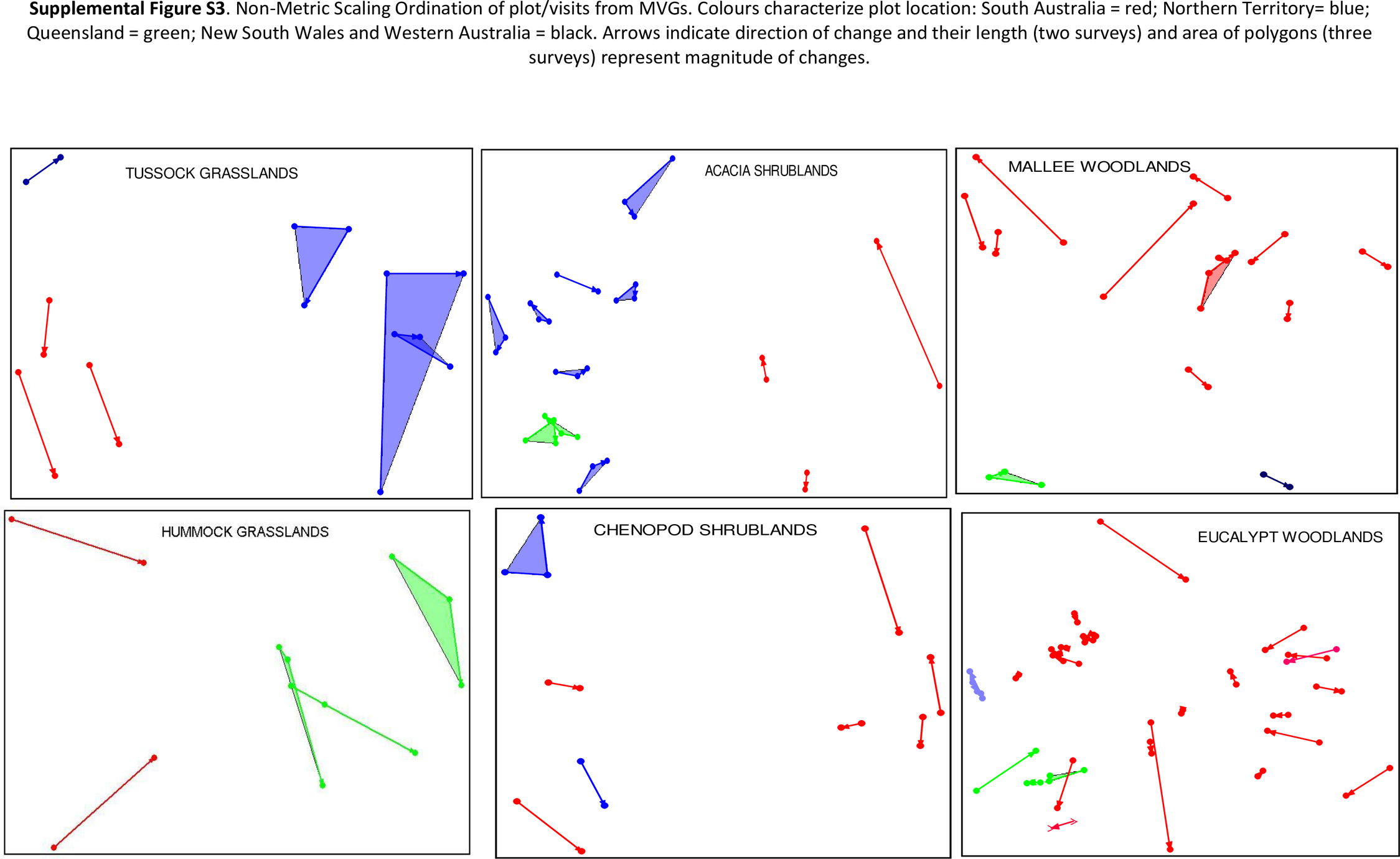

Supplement: S3 Fig — Colours characterize plot location: South Australia = red; Northern Territory = blue; Queensland = green; New South Wales and Western Australia = black. Arrows indicate direction of change and their length (two surveys) and area of polygons (three surveys) represent magnitude of changes. (TIF) [file pone.0278833.s003.tif]

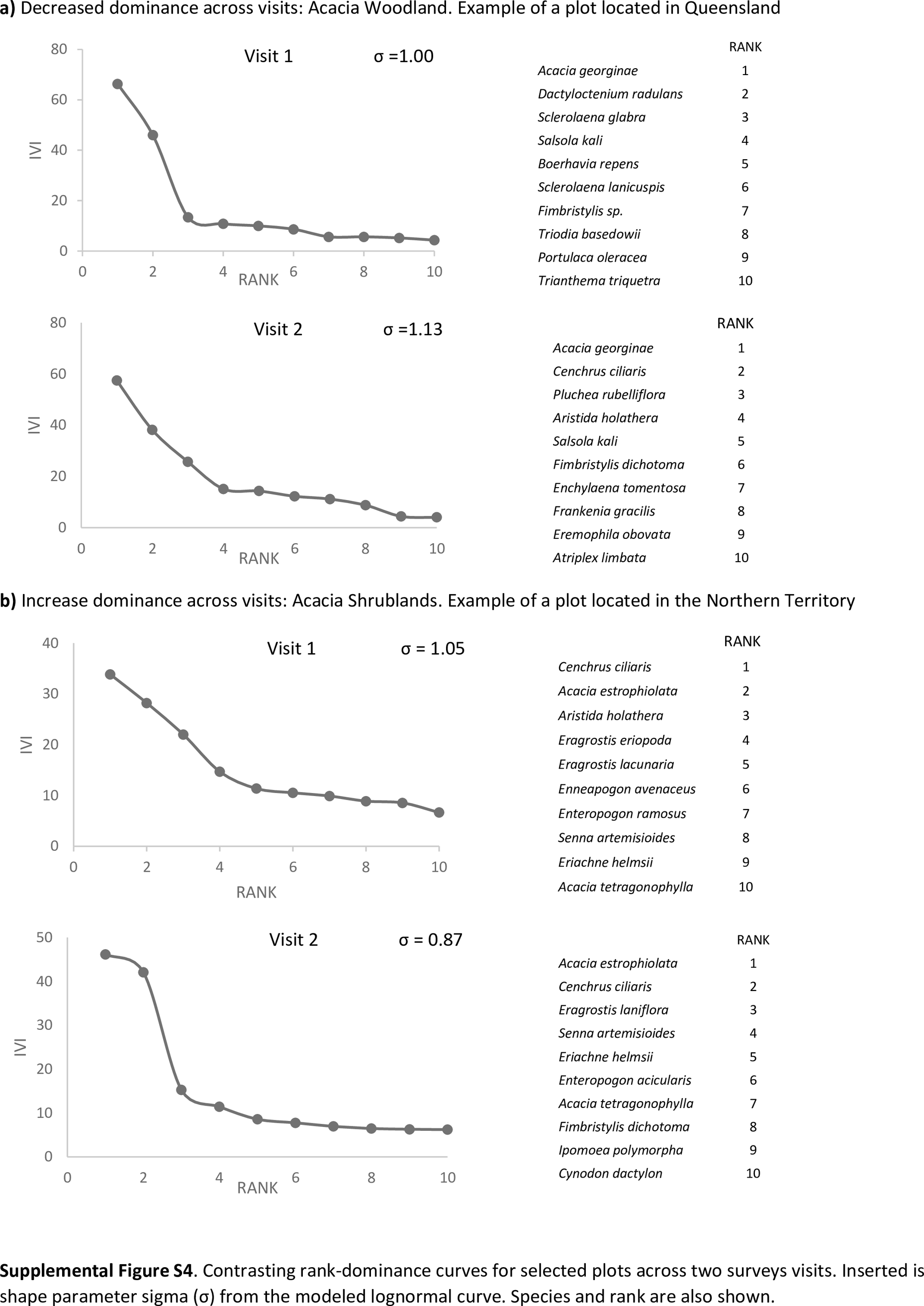

Supplement: S4 Fig — For selected plots across two surveys visits. Inserted is the shape parameter sigma (σ) from the modelled lognormal curve. Species and rank are also shown. (TIF) [file pone.0278833.s004.tif]

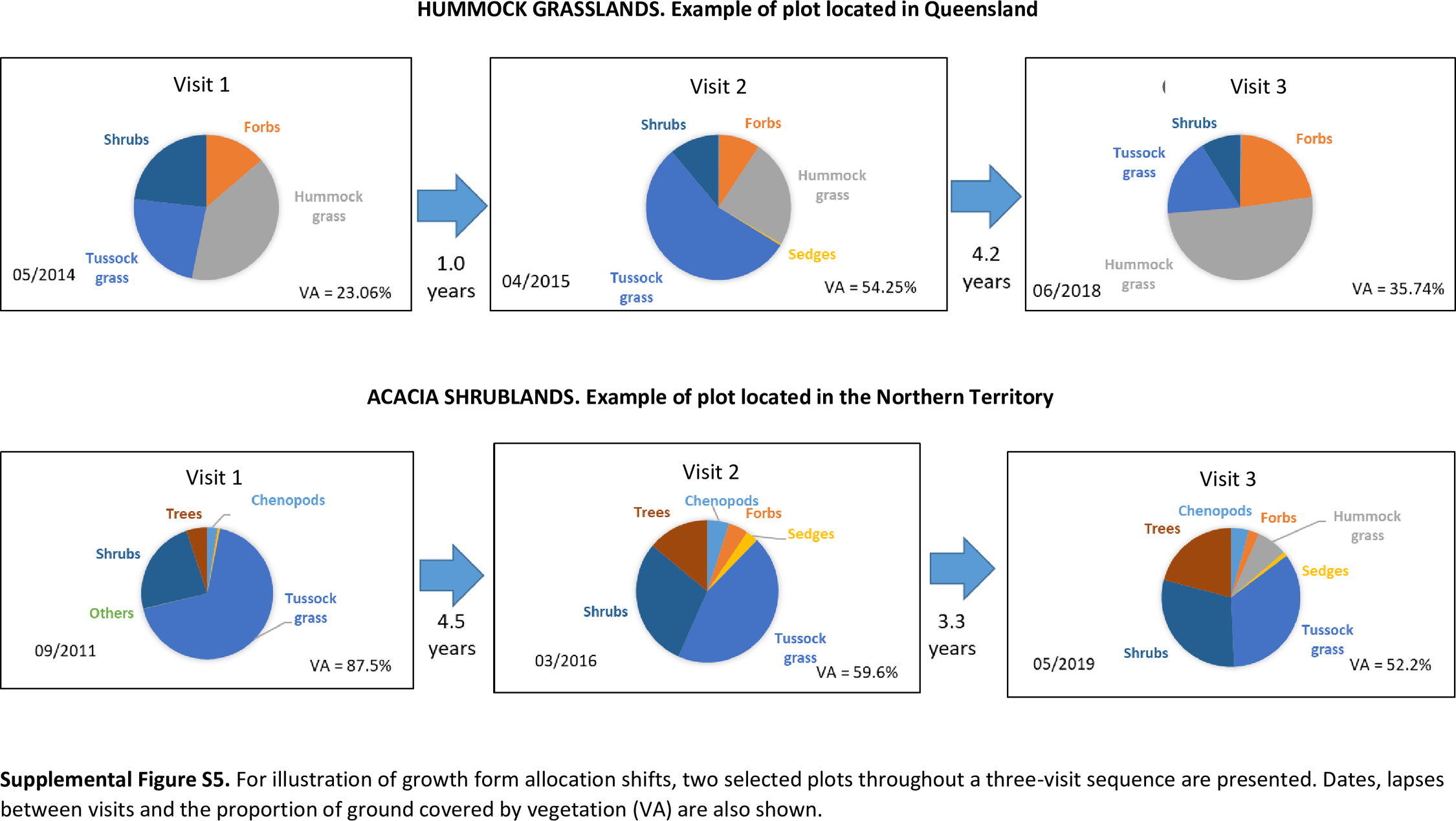

Supplement: S5 Fig — Two selected plots throughout a three-visit sequence are presented. Dates, lapses between visits and the proportion of ground covered by vegetation (VA) are also shown. (TIF) [file pone.0278833.s005.tif]
