# Supplementary material for: Shifts in floristic composition and structure in Australian rangelands
Source: PLoS One. 2022 Dec 14;17(12):e0278833. doi: 10.1371/journal.pone.0278833 (PMC9750033; doi:10.1371/journal.pone.0278833)
Supplement: S1 Table — Mean and standard deviation of main annual climatic parameters for Major Vegetation Groups. The Aridity Index is the ratio of precipitation to potential evaporation (higher values indicate less aridity). n = number sites. Data from Harwood et al. (2016). (DOCX) [file pone.0278833.s006.docx]

**Supplemental Table S1**. Mean and standard deviation of main annual climatic parameters for Major Vegetation Groups. The Aridity Index is the ratio of precipitation to potential evaporation (higher values indicate less aridity). n = number sites. Data from Harwood et al. (2016).

|  | Acacia Shrublands  (n=12) | Chenopod Shrublands  (n=8) | Hummock Grasslands  (n=5) | Tussock Grasslands  (n=7) | Woodlands  (n=46) |
| --- | --- | --- | --- | --- | --- |
| Aridity Index | 0.17 ± 0.07 | 0.17 ± 0.03 | 0.15 ± 0.05 | 0.14 ± 0.02 | 0.28 ± 0.21 |
| Potential evaporation (mm) | 1690.09 ± 205.26 | 1436.82 ± 157.57 | 1636.72 ± 296.92 | 1596.17 ± 193.40 | 1395.73 ± 255.74 |
| Mean Annual Precipitation (mm) | 283.19 ± 73.72 | 234.66 ± 42.60 | 235.87 ± 34.41 | 228.59 ± 55.31 | 353.19 ± 200.37 |
| Coefficient of Variation (%) | 26.03 | 18.15 | 14.59 | 24.19 | 57.73 |
| Maximum Temperature (°C) | 28.47 ± 2.72 | 25.99 ± 1.72 | 28.63 ± 4.58 | 28.11 ± 1.31 | 24.90 ± 3.91 |
| Minimum Temperature (°C) | 13.04 ± 1.86 | 11.76 ± 1.16 | 13.86 ± 3.18 | 13.06 ± 0.33 | 11.25 ± 2.46 |
| Temperature Range (°C) | 32.24 ± 1.99 | 30.02 ± 2.15 | 31.53 ± 1.36 | 32.22 ± 1.46 | 28.32 ± 3.41 |

*** Mean annual rainfall ranges four-fold: 200.3 - 979.9 mm.
