# Supplementary material for: Shifts in floristic composition and structure in Australian rangelands
Source: PLoS One. 2022 Dec 14;17(12):e0278833. doi: 10.1371/journal.pone.0278833 (PMC9750033; doi:10.1371/journal.pone.0278833)
Supplement: S2 Table — Indicated are the number of plots (n) and species. S, E, H’ and D designate plots species richness, equity, Shannon’s and Simpson diversity indices, respectively. Vouchered species refers to their total number within plots. Maximum Importance Value Index (IVI) denotes the highest value for species in the plots. Maximum IVI denotes increased dominance. β-Diversity indices by Whittaker’s (W) and as half-changes. (DOCX) [file pone.0278833.s007.docx]

**Supplemental Table S2.** Floristic metrics parameters and statistics of Major Vegetation Groups indicating number of plots (n) and species. S, E, H’ and D designate plots species richness, equity, Shannon’ and Simpson diversity indices, respectively. Vouchered species refers to their total number within plots. Maximum Importance Value Index (IVI) denotes the highest value for species in the plots. Maximum IVI denotes increased dominance. β-Diversity indices by Whittaker’s (W) and as half-changes (1/2).

| MAJOR VEGETATION GROUPS | S | E | H’ | D | Vouchered species | Maximum IVI | β-Div. (W) | β-Div. (1/2) |
| --- | --- | --- | --- | --- | --- | --- | --- | --- |
| Hummock Grasslands  n=13; 108 spps | 15.70 ± 4.96 | 0.69 ± 0.08 | 1.89 ± 0.39 | 0.71 ± 0.10 | 32.31 ± 10.13 | 98.39 ± 23.68 | 5.5 | 2.0 |
| Tussock Grasslands  n=17; 156 spps | 19.6 ± 10.62 | 0.82 ± 0.04 | 2.31 ± 0.56 | 0.84 ± 0.09 | 39.52 ± 20.03 | 58.4 ± 24.16 | 6.6 | 3.1 |
| Acacia Shrublands  n=31; 206 spps | 17.60 ± 5.64 | 0.79 ± 0.06 | 2.23 ± 0.37 | 0.83 ± 0.08 | 36.06 ± 9.66 | 59.72 ± 22.06 | 10.1 | 2.6 |
| Chenopod Shrublands  n=17; 151 spps | 17.5 ± 11.33 | 0.81 ± 0.06 | 2.23 ± 0.55 | 0.83 ± 0.08 | 36.00 ± 24.57 | 54.44 ± 22.16 | 7.1 | 2.8 |
| Eucalypt Woodlands  n=61; 458 spps | 21.08 ± 13.53 | 0.80 ± 0.06 | 2.29 ± 0.58 | 0.84 ± 0.08 | 42.44 ± 22.73 | 57.75 ± 21.40 | 21.3 | 3.7 |
| Mallee Woodlands  n=28; 271 spps | 19.71 ± 8.17 | 0.81 ± 0.05 | 2.35 ± 0.44 | 0.86 ± 0.07 | 39.25 ± 13.70 | 52.44 ± 19.86 | 12.1 | 3.3 |
