# Supplementary material for: Shifts in floristic composition and structure in Australian rangelands
Source: PLoS One. 2022 Dec 14;17(12):e0278833. doi: 10.1371/journal.pone.0278833 (PMC9750033; doi:10.1371/journal.pone.0278833)
Supplement: S1 Text — The IVI was calculated using the relative cover [(cover/summed cover for all species/growth forms) *100] and relative frequency [(frequency/summed frequency for all species/growth forms) *100] were calculated. Adding relative species cover and frequency generates the species IVI. Similarly, the IVI of each growth form was assessed by adding their relative number of species and its relative number of PIs. Species richness and Shannon and Simpson indices assess species diversity and relative dominance. The Sörensen (Bray-Curtis) distance and the Simpson Beta metrics [36], represent compositional change over time, including and excluding, respectively, differences in species richness. To contrast the rate of floristic shifts between MVGs on a common foundation, we annualised the shift of Sörensen distances by dividing them by the duration of the interval between visits and ANOVA tested for global differences among MVGs. In addition, to illustrate the extent of temporal floristic changes, plots from each MVG were ordered with the non-metric multidimensional scaling method (NMDS) representing Sörensen distances between visits as vectors [PC-Ord v7 [38]] To numerically contrast shifts in species dominance, we fitted lognormal models to the empirical species abundance distributions [39] then computed and tested the differences in the sigma (σ) shape parameter. The eight groups [31] for structural analysis are: (a) Trees (comprising Tree Mallee, Tree-Palm and similar); (b) Shrubs (including Heath Shrub, Shrub Mallee, and Acacia Shrub); (c) Sedges; (d) Hummock Grass; (e) Tussock Grass; (f) Chenopods; (g) Forbs and Herbs and (h) Others with minute abundance (Epiphyte, Fern, Rush, and Vines). Then, we computed their respective IVIs and determined their percentage within each MVG. (DOCX) [file pone.0278833.s008.docx]

**Supplemental S1 Text.** Expanded information on Data Management and Analysis

The IVI was calculated using the relative cover [(cover/summed cover for all species/growth forms) ∗100] and relative frequency [(frequency/summed frequency for all species/growth forms) ∗100] were calculated. Adding relative species cover and frequency generates the species IVI. Similarly, the IVI of each growth form was assessed by adding their relative number of species and its relative number of PIs.

Species richness and Shannon and Simpson indices assess species diversity and relative dominance. The Sörensen (Bray-Curtis) distance and the Simpson Beta metrics [36], represent compositional change over time, including and excluding, respectively, differences in species richness. To contrast the rate of floristic shifts between MVGs on a common foundation, we annualised the shift of Sörensen distances by dividing them by the duration of the interval between visits and ANOVA tested for global differences among MVGs. In addition, to illustrate the extent of temporal floristic changes, plots from each MVG were ordered with the non-metric multidimensional scaling method (NMDS) representing Sörensen distances between visits as vectors [PC-Ord v7 [38]] To numerically contrast shifts in species dominance, we fitted lognormal models to the empirical species abundance distributions [39] then computed and tested the differences in the sigma (σ) shape parameter.

The eight groups [31] for structural analysis are: (a) Trees (comprising Tree Mallee, Tree-Palm and similar); (b) Shrubs (including Heath Shrub, Shrub Mallee, and Acacia Shrub); (c) Sedges; (d) Hummock Grass; (e) Tussock Grass; (f) Chenopods; (g) Forbs and Herbs and (h) Others with minute abundance (Epiphyte, Fern, Rush, and Vines). Then, we computed their respective IVIs and determined their percentage within each MVG.
